# Effect of age on bone mineral density and micro architecture in the radius and tibia of horses: An Xtreme computed tomographic study

**DOI:** 10.1186/1746-6148-4-3

**Published:** 2008-01-25

**Authors:** A Fürst, D Meier, S Michel, A Schmidlin, L Held, A Laib

**Affiliations:** 1 Equine Hospital, Vetsuisse Faculty, University of Zurich, 8057 Zurich, Switzerland; 2 Swiss Federal Laboratories for Materials Testing and Research (Empa), 8600 Dübendorf, Switzerland; 3 Biostatistics Unit, Institute for Social and Preventive Medicine, University of Zurich, 8057 Zurich, Switzerland; 4 SCANCO Medical AG, 8303 Bassersdorf, Switzerland

## Abstract

**Background:**

The effect of age on the bone mineral density and microarchitecture of the equine radius and tibia was investigated. Fifty-six bones from 15 horses aged four to 21 years were used. There were nine geldings and six mares, and none of the horses had any disease influencing bone properties. Xtreme computed tomography was used to evaluate a 9-mm segment of the diaphysis and metaphysis of each bone. The following variables were determined: length of the bone, circumference and diameter in the frontal and sagittal planes in the middle of the bone.

Diaphysis: total volume, bone volume, bone volume ratio, slice area, bone area, marrow area, cortical and marrow thickness, bone mineral density, polar moment of inertia of the cortex.

Metaphysis: total area, bone area, cortical bone area, cortical thickness, bone mineral density, bone mineral density in the cortex, bone mineral density in the trabecular region, trabecular number, trabecular thickness, trabecular separation, polar moment of inertia of the metaphysis, polar moment of inertia of the cortex of the metaphysis.

**Results:**

Bone density and microarchitecture were not affected by breed or gender. However, the microarchitecture varied with the age of the horse; the number of trabeculae decreased significantly and the distance between trabeculae increased significantly with increasing age. There were no significant differences between bones of the left and right limbs or between the radius and tibia.

**Conclusion:**

The variables investigated did not differ between geldings and mares. However, there were age-related changes in the microstructure of the bones. Further experimental studies are necessary to determine whether these changes reduce bone strength. Age-related changes in the bones were seen and may explain the higher incidence of fractures and fissures in older horses.

## Background

**Figure 1:**
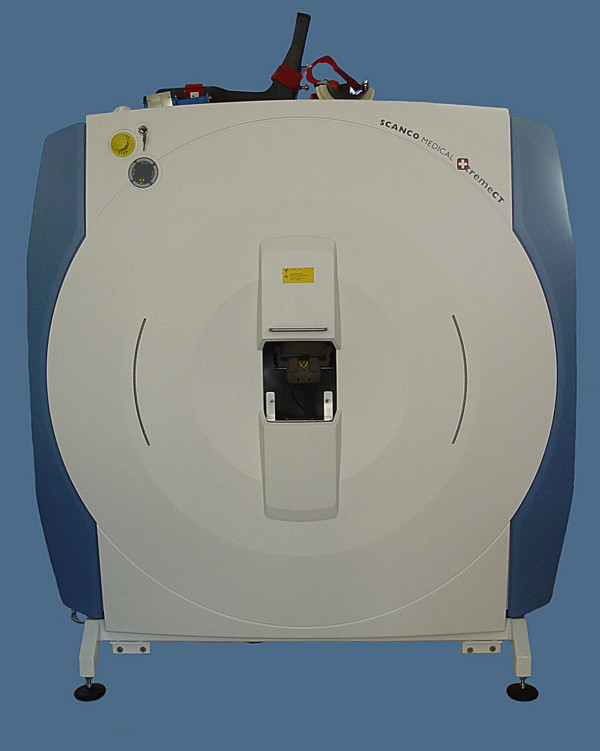
XtremeCT.

The ultimate strength of bone is determined by the bone mineral density (BMD) of the cortical and cancellous bone as well as the trabecular and cortical microarchitecture. In human medicine, determination of these variables is critical for the early detection of osteoporosis and other bone diseases. For years only BMD was determined, but recently three-dimensional microarchitecture was added to the list of criteria [1-3]. This is important because the BMD of healthy and osteoporotic bones can overlap [4,5]. Bone mineral density can be measured *in vivo *using dual energy x-ray absorptiometry (DEXA), quantitative ultrasonography (QUS) and peripheral quantitative computed tomography (pQCT) [1,6-9]. The three-dimensional bone structure can only be assessed using histomorphometric methods [6,9-12]. However, because trabecular bone consists of a three-dimensional network, even stereological techniques are not sufficient to produce an exact three-dimensional definition of the bone micro-structure based on histomorphological findings. The introduction of micro-computed tomography (micro-CT) allowed, for the first time, the stereological in-vivo examination of bone [8]. The decreasing number and thickness of bone trabeculae, but also other variables of bone architecture, can be detected earlier with micro-CT than via histomorphometry. In other studies age-related variations in the microstructure, the structure model type and trabecular thickness of human cancellous bone were investigated [10,13]; these studies showed that the Structure Model Index (SMI) increased and the trabecular thickness decreased with age. The SMI is defined as a value between 0 and 3 and is calculated from the relative amount of the number of trabecelae, which have the shape of rods or plates. Xtreme computed tomography (XtremeCT, Scanco Medical, Auenring 6–8, 8303 Bassersdorf, Switzerland), which is a high-resolution pQCT, provides micro-CT for the clinical diagnosis of osteoporosis and other bone diseases in human medicine. The resolution is limited to about 100 μm because of the low x-ray dose allowed for patients. The Xtreme-CT-generated structural data were verified by comparing them to values obtained using 28-micron-resolution micro-CT [14]. The correlation coefficients for the values obtained by the two methods ranged from 0.81 to 0.98. Laib and co-workers (1998) evaluated healthy post-menopausal women for microstructural changes using a prototype of Xtreme-CT (Fig. 1) and found that the extent as well as the localization of bone loss varies greatly among individuals [15].

Although equine bone diseases cannot be compared directly with osteoporosis in humans, they affect the use and longevity of the horse and therefore require adequate diagnosis and treatment. The most commonly-used diagnostic methods in horses include macroscopic and radiographic evaluation [16,17], DEXA and QUS. Dual energy x-ray absorptiometry has been used by Firth and co-workers (1999) to assess the effect of age, exercise and growth rate on osteochondrosis in foals [18]. McClure and co-workers (2001) compared BMD obtained via DEXA with the density that was measured using Archimedes' principle [19]. Donabedian and co-workers (2005) were the first to make DEXA measurements in live horses [20]. Quantitative ultrasonography is another commonly used diagnostic technique, and has the advantage of providing information other than BMD. This is because the attenuation of the sound waves (broadband ultrasound attenuation [BUA]) and the speed of sound through the bone (speed of sound [SOS]), two variables which are measured by QUS, are greatly affected by the mechanical properties and structure of the medium [21,22]. Quantitative ultrasonography has been used by Buckingham and Jeffcott (1991) to investigate the osteopenic effects of limb immobilization in horses [23,24]. Carstanjen and co-workers (2002) concluded that QUS was useful for the assessment of the metacarpus, radius and tibia in horses [25]. Another study investigated DEXA and QUS in horses and found that the two methods provided different information; QUS measured not only BMD but also the microstructure and composition of the bone [26].

Peripheral quantitative computed tomography is another non-invasive and very exact method of bone evaluation, which allows, to a certain extent, separate assessment of cortical and cancellous bone. The results of BMD determined by pQCT are in good agreement with other methods of measurement [27]. Cornelissen and co-workers (1999) used pQCT (XCT 960A, Stratec, Germany) to determine the influence of exercise on BMD of immature bone tissue in horses. Using a manually determined cut-off value, those authors assigned BMD values to trabecular bone. However, this did not constitute a direct measurement as is obtained via micro-CT. Computed tomography was also used by Waite and others [28] and Mäule and Gerhards (2004) to examine the distal limb of horses [29]. They concluded that the tissue density cannot be used as the sole criterion for the assessment of bone, because individual variations were too large. In addition to all the imaging techniques, biochemical markers can also be used to make an overall assessment of skeletal condition [21,30]. However, the methods described thus far allow only a general survey of the entire skeleton rather than the evaluation of individual bones. Furthermore, it has not been feasible to adequately and directly assess the microstructure of trabecular bone. The technique required to achieve this is micro-CT, which has rarely been used in horses [31,32].

In the present study, micro-CT (Xtreme CT) was used to obtain detailed data on cortical and trabecular bone, including microstructure, in the radii and tibiae of 15 horses. Special emphasis was given to the effects of age on the BMD and microarchitecture of the bones. These variables are likely to affect the mechanical properties of bones and may influence the fracture tendency in human bones [33]. Our investigations, together with more biomechanical testing, could provide better information as to whether changes in the microarchitecture influence the susceptibility to fractures. Likewise, the micro-CT data could provide the basis for using Finite Element Methods, which serve to determine the effects of various loads on bone and to simulate possible fracture configurations via a computer. The objective of this study was to investigate age-related changes in the BMD of the radius and tibia of the horse.

## Methods

Fifty-six bones, which consisted of 28 radii and 28 tibiae, of 15 horses euthanised at our clinic for various reasons (Table 1) between March 2001 and August 2004 were used. There were nine geldings and six mares, which ranged in age from four to 21 years. There were two thoroughbreds, two Franches-Montagnes and 11 warmblood horses. Generalized bone disease or other diseases that could influence bone properties were not diagnosed in any of the horses. Fourteen horses had been regularly used for low level competition or pleasure riding and one mare was used for breeding. Two tibiae and two radii were damaged during preparation. Immediately after euthanasia of the animals, the skin of the limbs was removed and the bones procured. The specimens were wrapped in cloth soaked in isotonic saline solution, and frozen at -20°C until further use. The specimens were allowed to thaw for one week before evaluation. Soft tissue, such as muscle, tendon, ligament and joint capsule, were removed. Then the part of the ulna that extended beyond the radius proximally, and the fibula, were removed with a saw (Bandsaw Type K 410, Kolbe GmbH, Elchingen, Germany). The original length was between 36.6 and 45.2 cm and the midpoint (50% mark) was marked. The point at the distal metaphysis (80% mark) that separated the proximal 80% from the distal 20% of the entire length was also marked. All bones had to be shortened to 38 cm to make them suitable for micro-CT scanning. To achieve this, the same lengths of bone were cut from the proximal and distal epiphyses so that the 50% mark remained exactly at the centre. At the 50% mark, the circumference and diameter of the bone in the frontal and sagittal planes were measured using a measuring tape.

**Table 1:** Gender, age, breed, use and reason for euthanasia in 15 horses used for Xtreme CT of the radius and tibia

Case number	Age in years	Sex	Breed	Use	Reason for euthanasia
1	20	gelding	Swiss warmblood	pleasure	cardiac disease
2	14	gelding	Hungarian warmblood	competition	kissing spines
3	10	mare	Irish warmblood	competition	back problem
4	21	gelding	Thorouthbred	pleasure	ruptured ligament
5	17	gelding	Swiss warmblood	pleasure	trauma
6	4	mare	Swiss warmblood	pleasure	epiglottic entrapment
7	11	mare	Swiss warmblood	pleasure	sarcoids
8	9	gelding	Hannovarian warmblood	competition	hoof abscess
9	10	mare	Wuerttemberg warmblood	pleasure	behavioral problem
10	15	gelding	Dutch warmblood	pleasure	colic
11	7	mare	Thorouthbred	pleasure	melanoma
12	18	gelding	Dutch warmblood	pleasure	colic
13	11	gelding	Franches-Montagnes	pleasure	colic
14	14	mare	Franches-Montagnes	blood mare	intoxication
15	15	gelding	Swiss warmblood	pleasure	ruptured tendon

Both ends of the bones were embedded in epoxy resin (Biresin^® ^G28 Harz and Biresin^® ^G26 Härter, 1:1 mixture, Sika Germany GmbH, Bad Urach, Germany) to a level above the widest part of the epiphysis, and the bones were labeled. The bones were re-wrapped in saline-soaked cloth, placed individually in plastic bags to prevent loss of moisture and stored at 4°C until further use.

**Figure 2:**
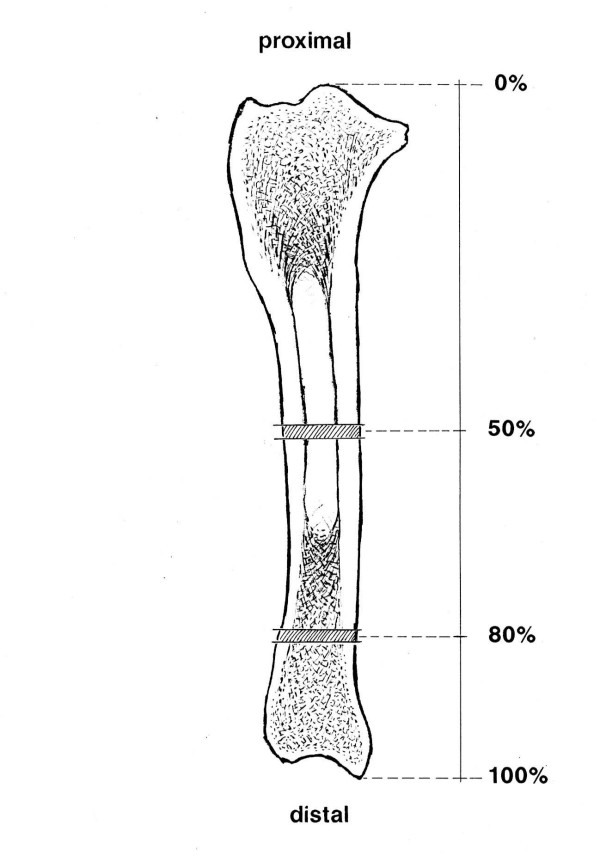
Diaphyseal and metaphyseal regions of the radius that were examined using XtremeCT.

A total of 56 bones were examined using XtremeCT (Fig. 1), which is a high resolution (41–246 μm nominal isotropic resolution) peripheral computed tomography unit for in-vivo measurement of BMD and microstructure in humans. In human medicine, this recently introduced imaging modality is used mainly for the early detection and monitoring of osteoporosis and other bone diseases in the distal radius and distal tibia. XtremeCT has a microfocused x-ray beam with a maximum scanning length of 150 mm. The scan-time for 9 mm (110 slices) is 3 minutes. The bones were scanned at the 50% mark (73 slices, resolution 123 μm) and at the 80% mark (110 slices, resolution 82 μm; Fig. 2). A computer software program (HP AlphaStation) was used to extract the BMD and microstructure data of the bones from the images.

XtremeCT measures a large number of geometric, densitometric and physical variables, which are displayed in Excel data sheets. All BMDs were expressed as mg hydroxyapatite/cm^3 ^(HA/cm^3^). The following variables were determined:

### In the diaphysis

Capitals for total and bone :  Total volume (in mm^3^),  Bone volume (in mm^3^), Slice Area = Total Area (in mm^2^), Bone Area (in mm^2^), Marrow Area (in mm^2^), Bone Volume Ratio, Cortical Thickness (in mm), Marrow Thickness (in mm), Bone Mineral Density (in mg HA/cm^3^), Polar moment of inertia of the cortex (in mm^4^).

### In the metaphysis

Total Area (in mm^2^), Bone Area (in mm^2^), Cortical Bone Area (in mm^2^) Cortical Thickness, Bone Mineral Density in the metaphysis (in mg HA/cm^3^), Bone Mineral Density in the cortex (in mg/cm^3 ^HA), Bone Mineral Density in the trabecular region (in mg HA/cm^3^), Trabecular Number, Trabecular Thickness, Trabecular Separation, Polar moment of inertia of the metaphysis, Polar moment of inertia of the cortex of the metaphysic

### Statistical analysis

A commercial software program was used for all calculations (SPSS, version 13 for Macintosh, SPSS Inc., Chicago). Statistical regression analysis was done using the software R [34]. Means and standard deviations were calculated for all variables. Measurements from the radii and tibiae were compared within horses using a paired *t*-test and the unpaired t-test was used to compare the bones between the horses. Linear regression analysis was used to determine the effect of age on the following outcome variables: DSlArea, DBoArea, DBMD, DpMOI, MD100, MCdens, MTb.N, MTb.Th, MTb.Sp, MpMOI, MDtrab. Type of bone (tibia or radius) was also included as an explanatory variable. To allow for possible correlations between observations in the same horse, a random effect was introduced for each horse [35]. The 5% significance threshold was adjusted for multiple testing, since 11 different outcomes were investigated. A corresponding Bonferroni correction yielded a threshold of 0.05/11 = 0.0045. A residual analysis indicated potential problems with heteroscedastic errors that increased with age. The model was extended to allow for dependence between the residual variance and age.

## Results

**Figure 3:**
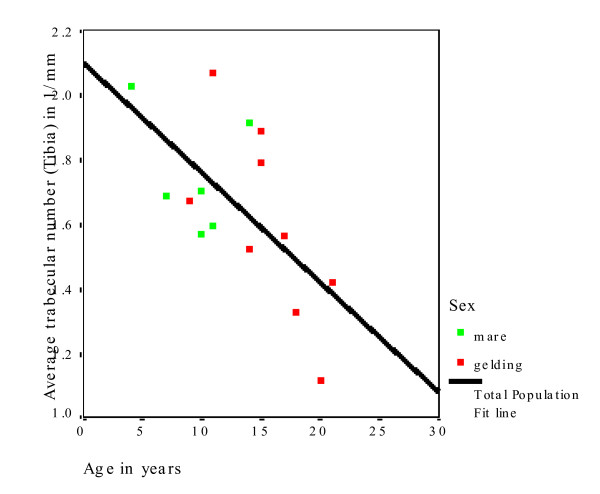
Decrease in the trabecular number in the tibia with increasing age of the horse.

**Figure 4:**
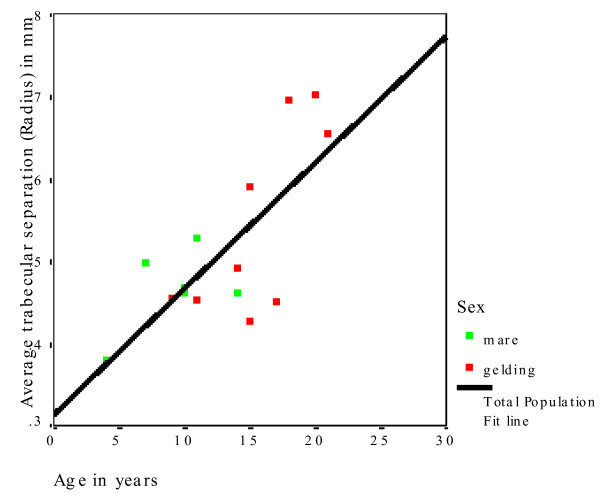
Increase in trabecular separation in the radius with increasing age of the horse.

XtremeCT provided very useful data about the macro- and micoarchitecture of the equine radius and tibia (Additional file 1). The mean slice area of the diaphysis was significantly smaller (P = 0.019) in the radius (1,468 mm^2^) than in the tibia (1,699 mm^2^) (Table 2). The mean cortical thickness of the diaphysis was significantly smaller (P < 0.001) in the radius (9.97 mm) than in the tibia (11.33 mm; Table 3). There was no significant difference between the cortical thickness of mares and geldings. Horses in the middle age group had numerically higher means for both the radius and tibia than horses in the younger and older age groups, but differences were not statistically significant. The mean BMD of the cortex of the radius was 1,179 mg HA/cm^3^. Age and gender of the horses had no effect on BMD of the radius. The mean BMD of the cortex of the tibia was 1,175 mg HA/cm^3^. Age and gender of the horse had no effect on the diaphyseal BMD of the tibia (Table 4). The mean slice area of the metaphysis of the radius was 2,026 mm^2 ^and that of the tibia was 1,981 mm^2^, and there was no effect of age and gender on these variables (Table 5). For both bones, the younger horses had the numerically highest values. The mean cortical thickness of the metaphysis was significantly (P < 0.001) smaller in the radius (4.12 mm) than in the tibia (5.06 mm; Table 6). There was no significant effect of age on this variable, although the horses of the middle age group had the numerically highest values. The mean trabecular number in the radius was 1.73/mm and in the tibia it was 1.65/mm (Table 7). There was no significant effect of gender on the trabecular number of either bone; however, age had a significant effect on this variable (Fig. 3). Horses in the oldest age group had significantly fewer trabeculae (radius, P = 0.002; tibia, P = 0.012) compared with the middle and youngest age group. The mean trabecular thickness was 0.077 mm in the radius and 0.079 mm in the tibia (Table 8). There was no significant effect of age and gender on this variable. The mean distance between individual trabeculae (trabecular separation) was 0.514 mm in the radius and 0.541 mm in the tibia (Table 9). Trabecular separation increased significantly with increasing age in both the radius (P = 0.002) and tibia (P = 0.006; Fig. 4). Gender had no significant effect on trabecular separation in the radius or tibia.

**Table 2:** Slice area of the radial and tibial diaphyses

	Subgroup	Minimum	Maximum	Mean	Standard Deviation	N
Slice Area Diaphyse (mm^2^) Radius	total	1247.46	1782.49	1468.22	153.34	15
	geldings	1359.65	1782.49	1512.44	161.69	9
	mares	1247.46	1563.52	1401.88	123.30	6
	1–8 years old	1370.83	1563.52	1467.18	136.26	2
	9–16 years old	1247.46	1782.49	1470.85	168.82	9
	17–24 years old	1359.65	1709.46	1462.81	165.78	4
Slice Area Diaphyse (mm^2^) Tibia	total	1445.98	2150.56	1698.84	164.28	15
	geldings	1615.22	2150.56	1746.31	176.06	9
	mares	1445.98	1753.05	1627.63	125.81	6
	1–8 years old	1711.57	1753.05	1732.31	29.33	2
	9–16 years old	1445.98	2150.56	1688.58	204.63	9
	17–24 years old	1615.22	1855.74	1705.18	113.49	4

**Table 3:** Cortical thickness of the radius and tibial diaphyses

	Subgroup	Minimum	Maximum	Mean	Standard Deviation	N
Cortical Thickness Diaphyse (mm) Radius	total	8.44	13.60	9.97	1.41	15
	geldings	8.44	13.60	10.19	1.65	9
	mares	8.63	11.50	9.63	1.00	6
	1–8 years old	9.00	9.44	9.22	0.31	2
	9–16 years old	8.63	13.60	10.52	1.55	9
	17–24 years old	8.44	10.23	9.11	0.78	4
Cortical Thickness Diaphyse (mm) Tibia	total	9.93	13.01	11.33	0.97	15
	geldings	9.93	13.01	11.36	1.15	9
	mares	10.44	12.19	11.29	0.72	6
	1–8 years old	10.59	11.00	10.80	0.29	2
	9–16 years old	10.44	13.01	11.78	0.99	9
	17–24 years old	9.93	11.00	10.59	0.49	4

**Table 4:** Bone mineral densitiy of the radial and tibial diaphysis

	Subgroup	Minimum	Maximum	Mean	Standard deviation	N
Bone Mineral Density (Cortex, mg HA/cm^3^) Radius	total	1149.37	1220.57	1178.76	24.23	12
	geldings	1149.37	1220.57	1180.94	28.86	6
	mares	1150.09	1214.10	1176.58	21.15	6
	1–8 years old	1150.09	1176.52	1163.30	18.69	2
	9–16 years old	1149.37	1214.10	1180.72	23.75	6
	17–24 years old	1150.18	1220.57	1183.56	29.97	4
Bone Mineral Density (Cortex, mg HA/cm^3^) Tibia	total	1122.40	1215.27	1174.96	26.72	11
	geldings	1122.40	1204.30	1172.44	30.43	6
	mares	1149.91	1215.27	1177.98	24.64	5
	1–8 years old	1149.91	1149.91	1149.91	0.00	1
	9–16 years old	1122.40	1215.27	1176.89	32.14	6
	17–24 years old	1157.91	1204.30	1178.32	20.67	4

**Table 5:** Slice area of the radius and tibial metaphyses

	Subgroup	Minimum	Maximum	Mean	Standard Deviation	N
Slice Area Metaphyse (mm^2) ^Radius	total	1732.0	2531.5	2026.10	215.26	15
	geldings	1760.0	2348.0	2005.50	187.82	9
	mares	1732.0	2531.5	2057.00	267.18	6
	1–8 years old	2111.5	2531.5	2321.50	296.98	2
	9–16 years old	1732.0	2348.0	1951.33	195.81	9
	17–24 years old	1911.0	2116.0	2046.63	95.27	4
Slice Area Metaphyse (mm^2^) Tibia	total	1610.0	2328.0	1981.37	177.27	15
	geldings	1830.0	2328.0	2000.39	157.63	9
	mares	1610.0	2255.5	1952.83	215.88	6
	1–8 years old	2015.5	2255.5	2135.50	169.71	2
	9–16 years old	1610.0	2328.0	1964.06	202.27	9
	17–24 years old	1854.0	2030.0	1943.25	96.28	4

**Table 6:** Cortical thickness of the radial and tibial metaphyses

Cortical Thickness Metaphyse (mm) Radius	total	3.46	4.95	4.12	0.50	15
	geldings	3.47	4.95	4.30	0.53	9
	mares	3.46	4.29	3.86	0.33	6
	1–8 years old	3.51	4.29	3.90	0.55	2
	9–16 years old	3.46	4.95	4.30	0.50	9
	17–24 years old	3.47	4.36	3.83	0.39	4
Cortical Thickness Metaphyse (mm) Tibia	total	4.52	5.86	5.06	0.44	15
	geldings	4.56	5.86	5.09	0.43	9
	mares	4.52	5.77	5.01	0.50	6
	1–8 years old	4.54	5.77	5.15	0.87	2
	9–16 years old	4.52	5.86	5.18	0.41	9
	17–24 years old	4.56	4.84	4.74	0.13	4

**Table 7:** Trabecular number in radius and tibia

	Subgroup	Minimum	Maximum	Mean	Standard-deviation	N
Trabecular number (1/mm) Radius	total	1.27	2.12	1.73	0.26	15
	geldings	1.27	2.00	1.66	0.30	9
	mares	1.65	2.12	1.85	0.15	6
	1–8 years old	1.79	2.12	1.95	0.23	2
	9–16 years old	1.53	2.00	1.80	0.15	9
	17–24 years old	1.27	1.94	1.47	0.32	4
Trabecular number (1/mm) Tibia	total	1.12	2.07	1.65	0.26	15
	geldings	1.12	2.07	1.59	0.29	9
	mares	1.57	2.03	1.75	0.18	6
	1–8 years old	1.69	2.03	1.86	0.24	2
	9–16 years old	1.52	2.07	1.74	0.18	9
	17–24 years old	1.12	1.56	1.35	0.19	4

**Table 8:** Trabecular thickness

	Subgroup	Minimum	Maximum	Mean	Standard-deviation	N
Trabecular Thickness (mm) Radius	total	0.062	0.095	0.077	0.01	15
	geldings	0.062	0.095	0.077	0.01	9
	mares	0.063	0.095	0.079	0.01	6
	1–8 years old	0.063	0.095	0.079	0.02	2
	9–16 years old	0.062	0.095	0.077	0.01	9
	17–24 years old	0.068	0.086	0.078	0.01	4
Trabecular Thickness (mm) Tibia	total	0.056	0.102	0.079	0.01	15
	geldings	0.061	0.102	0.078	0.01	9
	mares	0.056	0.098	0.081	0.01	6
	1–8 years old	0.056	0.098	0.077	0.03	2
	9–16 years old	0.061	0.093	0.077	0.01	9
	17–24 years old	0.078	0.102	0.086	0.01	4

**Table 9:** Trabecular separation of radius and tibia

	Subgroup	Minimum	Maximum	Mean	Standard Deviation	N
Trabecular Separation (mm) Radius	total	0.38	0.70	0.51	0.10	15
	geldings	0.43	0.70	0.55	0.11	9
	mares	0.38	0.53	0.47	0.05	6
	1–8 years old	0.38	0.50	0.44	0.08	2
	9–16 years old	0.43	0.59	0.48	0.05	9
	17–24 years old	0.45	0.70	0.62	0.12	4
Trabecular Separation (mm) Tibia	total	0.40	0.80	0.54	0.10	15
	geldings	0.42	0.80	0.57	0.11	9
	mares	0.40	0.55	0.50	0.07	6
	1–8 years old	0.40	0.54	0.47	0.10	2
	9–16 years old	0.42	0.56	0.50	0.05	9
	17–24 years old	0.56	0.80	0.66	0.10	4

**Figure 5:**
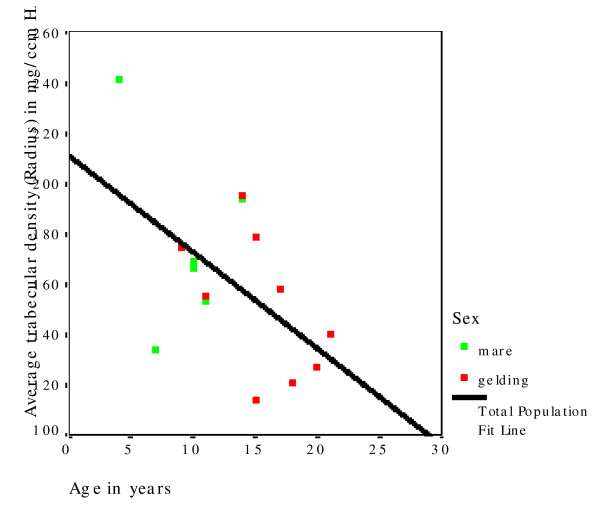
Decrease in trabecular density in the radius with increasing age of the horse.

The mean cortical BMD of the metaphysis was 1,035 mg HA/cm^3 ^in the radius and 1,049mg HA/cm^3 ^in the tibia (Table 10). This variable increased significantly with increasing age in both the radius (P = 0.028) and tibia (P = 0.047). Gender had no significant effect on the mean cortical BMD of the metaphysis. Thus, the mean cortical BMD of the diaphysis was significantly greater than the mean cortical BMD of the metaphysis in both the radius and tibia (P < 0.001). The mean trabecular BMD (entire trabecular region) was 161 mg HA/cm^3 ^in the radius and 156 mg HA/cm^3 ^in the tibia (Table 11). In the radius, this variable decreased significantly with increasing age (P = 0.033), and in the tibia there was a trend for a decrease with increasing age (P = 0.054; Fig. 5). Gender had no significant effect on trabecular BMD. The changes in trabecular BMD, number and separation observed in the horses of the oldest age group did not reach osteoporotic proportions. An initial regression analysis indicated that neither gender nor breed had significant effects on the different outcomes. Those variables were omitted from subsequent analyses. The effect of age on the different outcome variables is summarized in Table 12. Even after allowing for multiple testing, age had a significant effect on the two variables MTb.N and MTb.Sp. These findings did not change qualitatively after allowing for heteroscedasticity.

**Table 10:** Cortical density

	Subgroup	Minimum	Maximum	Mean	Standard Deviation	N
Cortical Density Metaphysis (mg HA/cm^3^) Radius	total	990.50	1074.00	1035.25	21.47	12
	geldings	1017.00	1074.00	1042.92	20.13	6
	mares	990.50	1053.00	1027.58	21.63	6
	1–8 years old	990.50	1016.00	1003.25	18.03	2
	9–16 years old	1017.00	1057.00	1038.83	14.48	6
	17–24 years old	1031.00	1074.00	1045.88	19.33	4
Cortical Density Metaphysis (mg HA/cm^3^) Tibia	total	980.00	1114.00	1048.55	35.95	11
	geldings	1027.00	1114.00	1062.08	13.56	6
	mares	980.00	1064.00	1032.30	37.16	5
	1–8 years old	980.00	980.00	980.00	0.00	1
	9–16 years old	1006.50	1064.00	1044.08	22.56	6
	17–24 years old	1041.50	1114.00	1072.38	33.11	4

**Table 11:** Trabecular density

	Subgroup	Minimum	Maximum	Mean	Standard Deviation	N
Trabecular Density (mg HA/cm^3^) Radius	total	114.00	240.50	161.13	33.19	15
	geldings	114.00	195.00	151.28	28.04	9
	mares	133.50	240.50	175.92	37.28	6
	1–8 years old	133.50	240.50	187.00	75.66	2
	9–16 years old	114.00	195.00	166.44	24.53	9
	17–24 years old	120.50	158.00	136.25	16.51	4
Trabecular Density (mg HA/cm^3^) Tibia	total	113.00	238.00	155.57	29.36	15
	geldings	135.00	169.00	145.67	12.33	9
	mares	113.00	238.00	170.42	41.58	6
	1–8 years old	113.00	238.00	175.50	88.39	2
	9–16 years old	138.00	191.00	159.00	15.90	9
	17–24 years old	135.00	145.50	137.88	5.11	4

**Table 12:** Regression analysis

outcome variables	Value	Std. Error	t-value	p-value
DSlArea	-2.20	8.80	-0.25	0.81
DBoArea	-3.39	7.48	-0.45	0.66
DBMD	-0.25	5.36	-0.05	0.96
MD100	-2.45	3.98	-0.62	0.55
MCdens	1.28	4.89	0.26	0.80
MTb.N	-0.04	0.01	-3.76	0.0024*
MTb.Th	0.00	0.00	0.32	0.76
MTb.Sp	0.01	0.00	3.89	0.0018*
DpMOI	-1873.91	4592.74	-0.41	0.69
MpMOI	-7762.35	4730.40	-1.64	0.12
MDtrab	-3.48	1.48	-2.35	0.035*

## Discussion

In the present study, XtremeCT, a type of peripheral computed tomography, was used to evaluate the microstructure of trabecular and cortical bone of the horse. This technique was originally designed to evaluate the human tibia and radius [15]. In contrast to human medicine, this technique is not suited for in-vivo studies in the horse because of certain restrictions. Extremities to be examined must remain in the Xtreme computed tomographic scanner without moving for six minutes. It would therefore be feasible theoretically to examine the third phalangeal bone in the anaesthetized horse. Soft tissue was removed from the bones in this study to allow solid fixation of the bones in the measuring apparatus (Fig. 1) and to identify the two predetermined measuring sites (Fig. 2). To date, there have only been a limited number of studies on microtomography in which a prototype of Xtreme-CT was used, and these studies dealt exclusively with human bone [11,15].

Our results showed that the equine tibia had a larger mean cross-sectional surface area and a thicker diaphyseal and metaphyseal cortex than the radius. However, there were no significant differences between the radius and the tibia with respect to the trabecular structure. A possible reason for this is that in the horse, but not in man, both the radius and tibia are weight-bearing bones. In humans, the microstructure of cancellous bone varies, particularly among long bones, vertebrae and the flat pelvic bones [36].

There were very few apparent differences between the bones of mares and geldings. However, studies using a larger number of horses, including stallions and mares of all age groups are needed to confirm our results. The effects of exercise and different riding disciplines on BMD and bone architecture should also be investigated. Training has a positive effect on bone micro-architecture [24,37,38]. In humans, the differences between the bone structure of young men and women are also minimal. Only the peak bone mass, which is the maximum bone mass, is markedly higher in men than in women [39]. This is because men are generally taller than women and thus have larger bones. There are distinct differences in the loss of bone mass of men and women as they age. Although an age-related loss of bone mass is seen in both genders, the loss in women is more rapid and marked in the first few years after menopause [40]. This predisposes older women to a higher incidence of fractures. The rapid loss in bone mass is attributable to a sudden decrease in estrogen concentration during menopause. Unlike women, aged mares do not undergo a precipitous decrease in estrogen concentration because they maintain follicular activity [41], and therefore, a significantly different bone microstructure from that seen in older geldings or stallions would not be expected. More studies are needed to investigate whether geldings have poorer bone quality than stallions because of lower levels of male sex hormones.

**Figure 6:**
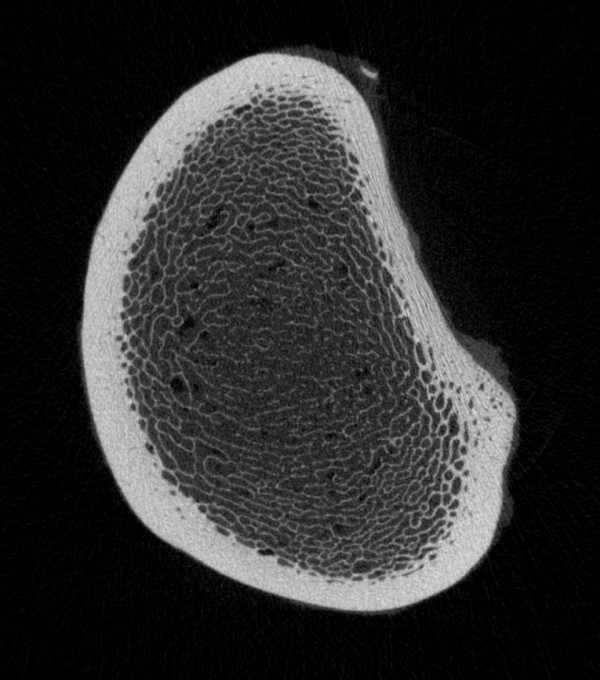
Radius with a small number of trabeculae.

**Figure 7:**
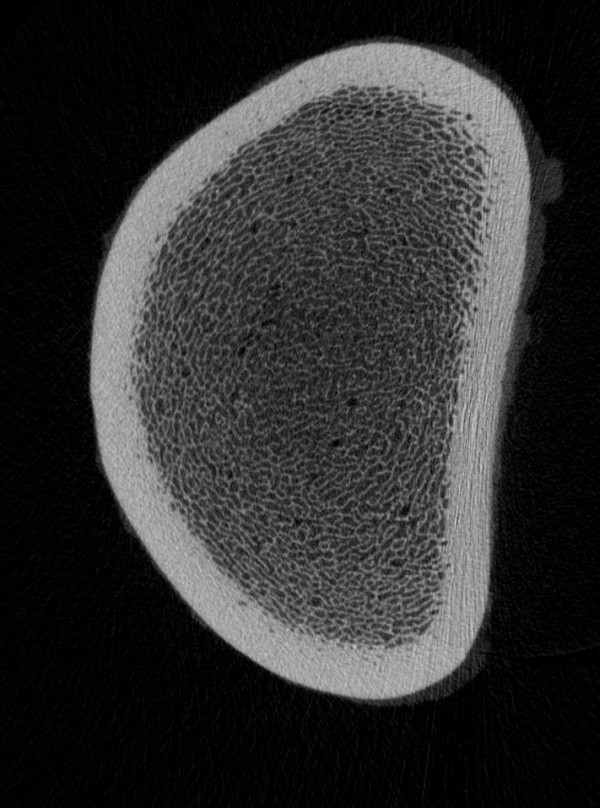
Radius with a large number of trabeculae.

With respect to the effect of age on bone quality, the trabecular number (Fig. 3 and 4) and trabecular BMD (Fig. 5) decreased, and the trabecular separation and cortical BMD of the metaphysis increased in the radius and tibia with increasing age. Based on these findings, we assume that the bone trabeculae are not replaced but rather progressively decrease in number with age. The trabecular thickness and volume also tended to decrease, although the changes were not significant. Evaluation of a larger number of bones may yield more age-related variables. Evaluation of the polar moment of inertia of the diaphysis and metaphysis of the radius and tibia revealed no age-related changes. Although in one study, horses with fractures were older than the overall equine patient population [42], there are no more indications that older horses have a higher incidence of fractures than younger horses. There is no scientific evidence of age-related osteoporosis in horses. Osteoporosis is defined as a severe loss in BMD predisposing the individual to spontaneous fractures [43]. Osteopenia, on the other hand, describes a decrease in BMD that is not associated with spontaneous fractures [44]. According to these definitions, the changes in trabecular BMD, number and separation that we observed in the older horses do not constitute osteoporosis (Fig. 6 and 7). Because of this, osteopenia commonly goes unnoticed and is probably more common than osteoporosis. However, other studies involving mostly cannon bones suggest that a decrease in the number and thickness of the trabeculae increases the risk of fracture [38-40]. Several studies have evaluated the mechanical properties of bone using micro-computed tomography and related these properties to bone strength [33,45-47]. Nevertheless, because they have been used with success in elderly people, locking compression plates, which increase the stability of fracture repair, might be advantageous in horses as well [48].

In humans, the effect of age varies with the type of bone; using histomorphometry, scanning electron microscopy and biomechanical testing, Mosekilde (2000) determined that the weight-bearing ability of the human vertebrae was approximately 1,000 kg in young adults, but only 150 to 250 kg in the elderly [40]. This change is attributable to a decrease in the trabecular BMD, trabecular bone volume ratio, ash density and cortical thickness. However, the trabecular thickness decreases only in certain bones, for example in the femur [10]. These changes are first seen in people over 80 years of age. The structure model index also changes from a plate-like to a rod-like structure [10,13], and the bone volume/total volume ratio as well as the bone strength decrease; however, these changes occur slightly earlier than thinning of the trabeculae and may be seen at the age of 60 [13].

Our study investigated BMD and microarchitecture of the radius and tibia because we have been interested in the frequency and configurations of fractures of the tibia and radius for some time [49]. Other studies have dealt with metacarpal and metatarsal bones of young racehorses [27] using DEXA, QUS or QCT. In another study, age-related changes of the mechanical properties of the metacarpal bones in horses were investigated based on changes in BMD, mechanical strength and fragility [50]. The BMD was not affected by the gender and the age of the horses, although it increased numerically until the age of six and then remained unchanged or decreased again. The strength of the bone peaked at about 4.5 years of age and correlated well with the BMD. The fragility of the bone peaked at about six years of age and thereafter was the only variable that decreased significantly with age.

## Conclusion

To our knowledge, there are no high-resolution imaging studies on the micro-architecture of equine long bones and how it is affected by the gender and age of the horse. The results of the present study show that bone microstructure undergoes age-related changes, which may predispose to fractures. Further investigations are necessary to determine the effect of microarchitectural changes on the strength of bones and their susceptibility to fracture. Such studies should include very old mares to investigate the effect of ovarian senescence on bone micro-architecture.

## Authors' contributions

AF conceived the idea for the study, was involved in the analysis of the data and wrote the manuscript. DM collected the data and was involved in analysis of the data. SM made substantial contributions to the design of the study and assisted in the interpretation of the data. AS assisted during examination of the bones using Xtreme-CT. LH carried out the statistical analysis. AL assisted in the analysis and interpretation of the data and was involved in the drafting of the manuscript

## Supplementary Material

Additional File 1 List of measurements Click here for file
