# Supplementary material for: Effect of age on bone mineral density and micro architecture in the radius and tibia of horses: An Xtreme computed tomographic study
Source: BMC Vet Res. 2008 Jan 25;4:3. doi: 10.1186/1746-6148-4-3 (PMC2267174; doi:10.1186/1746-6148-4-3)
Supplement: Additional File 1 — List of measurements [file 1746-6148-4-3-S1.doc]

**Table 2:** Measurements, Mean, Median, Highest and lowest value, Standard deviation
